# Health Risk Assessment of Ambient Air Concentrations of Benzene, Toluene and Xylene (BTX) in Service Station Environments

**DOI:** 10.3390/ijerph110606354

**Published:** 2014-06-18

**Authors:** Benjamin Edokpolo, Qiming Jimmy Yu, Des Connell

**Affiliations:** Griffith School of Engineering, Griffith University, Nathan Campus, Brisbane, Queensland, 4111, Australia; E-Mails: jimmy.yu@griffith.edu.au (Q.J.Y.); d.connell@griffith.edu.au (D.C.)

**Keywords:** exposure assessment, service station, health risk assessment, hazard quotient, cancer risk, overall risk probability

## Abstract

A comprehensive evaluation of the adverse health effects of human exposures to BTX from service station emissions was carried out using BTX exposure data from the scientific literature. The data was grouped into different scenarios based on activity, location and occupation and plotted as Cumulative Probability Distributions (CPD) plots. Health risk was evaluated for each scenario using the Hazard Quotient (HQ) at 50% (C_EXP50_) and 95% (C_EXP95_) exposure levels. HQ_50_ and HQ_95_ > 1 were obtained with benzene in the scenario for service station attendants and mechanics repairing petrol dispensing pumps indicating a possible health risk. The risk was minimized for service stations using vapour recovery systems which greatly reduced the benzene exposure levels. HQ_50_ and HQ_95_ < 1 were obtained for all other scenarios with benzene suggesting minimal risk for most of the exposed population. However, HQ_50_ and HQ_95_ < 1 was also found with toluene and xylene for all scenarios, suggesting minimal health risk. The lifetime excess Cancer Risk (CR) and Overall Risk Probability for cancer on exposure to benzene was calculated for all Scenarios and this was higher amongst service station attendants than any other scenario.

## 1. Introduction

Petrol vapour emissions constitute one of the main sources of air pollutants in service stations (Table 1). Petrol is a complex mixture consisting mainly of hydrocarbons with a range of 3–11 carbon atoms [1]. There are a wide range of Volatile Aromatic Hydrocarbons (VAHs) present in the atmosphere of service stations as a result of emissions of vapours during dispensing, loading, unloading and transportation of petrol [2]. The major VAHs are benzene, toluene and xylene, often referred to as the BTX compounds [3].

**Table 1:** Investigations of concentrations reported as individual values of BTX in service station environments.

Location	Description (BTX **)	Sampling Method *	Reference
France	BTX concentrations near a stage II implemented petrol station	S	[1]
Spain	Exposure from a group of filling station attendants (BTX)	P	[3]
Mexico	Personal exposure in service stations	P	[4]
Brazil	Impact of emissions from gas stations into the atmosphere (BTX)	S	[5]
Spain and Belgium	Measurements in the vicinity of petrol stations (BTX)	S	[6]
South Africa	Personal exposure in African petrol attendants (BTX)	P	[7]
Finland	Customer during gasoline refuelling (BTX)	S	[8]
Spain	Impact from petrol stations surroundings (B)	S	[9]
Taiwan	Investigation Aromatic Compound Concentration at a Gas Service Station (BT)	S	[10]
Spain	Assessing air quality inside vehicles and at filling stations by monitoring (BTX)	S	[11]
Finland	Exposure to Aromatic Hydrocarbons during gasoline pump maintenance, repair and inspection (BTX)	P	[12]
Spain	Environmental and biological monitoring volatile organic compounds in the workplace (BTX)	P	[13]
Europe (Belgium, France, Germany, Greece, Ireland, Italy, Norway, Portugal, Sweden)	A preliminary study of ambient air concentrations of benzene around service stations and distribution terminals in Europe (B)	S	[14]
United Kingdom	A year long study of ambient air concentrations of benzene around a service station (B)	S	[15]
United Kingdom	The Measurement of Benzene Concentrations in the Vicinity of Petrol Stations (B)	S	[16]

Notes: * P is personal sampling, S is static sampling; ** B: benzene, T: toluene, X: xylene.

Benzene is regarded as the most hazardous compound of the BTX group. The International Agency for Research Cancer (IARC) and United State Environmental Protection Agency (USEPA) have classified benzene to be a Group A and CLASS 1 human carcinogen respectively [17] but, toluene and xylene do not fall in this group [18,19]. Short term human exposures to relatively high concentrations of benzene can give rise to various adverse health effects such as headaches, dizziness, inability to concentrate, impaired short term memory and tremors [20]. While long term exposures can give rise to more complex health effects that include haematotoxicity, genotoxicity, immunological and reproductive effects as well as various cancers [7,17].

Health risk assessment for toxic pollutants can be carried out to evaluate the possible adverse effects of exposure to these substances [21,22,23]. Various studies have shown chronic exposures to benzene with service station attendants may result in the occurrence of cancer and other adverse health effects [24]. In a similar study, DNA damage was observed with service station attendants exposed to benzene [7]. Many studies on BTX exposure in service stations have been conducted and are summarized in Table 1. BTX concentrations in the air were reported to be highest for service station attendants as compared to other locations in and around the service stations. Investigations in Iran with service station attendants found they are more at risk of adverse health effects than drivers [25]. Another study in Thailand showed that service station attendants were at greater health risk than any other occupation from exposure to benzene [20].

Exposure to toxicants can be evaluated using guidelines based on the Acceptable Daily Intake (ADI), Minimal Risk Level (MRL) and Reference Dose (RfD) as single points to quantify the risk [26]. However, risk assessment using probabilistic techniques utilizes probability distributions to estimate the risk [27,28,29]. This technique gives a quantitative description of uncertainty and variability in evaluating the risk of health effects [30].

In previous studies, we have evaluated the health risk of chlorination disinfection by-products in drinking water using probabilistic techniques which suggested that there are a number of possible adverse health effects [28]. Also a study of the health risk due to chlorobenzenes in the air of residential houses using probabilistic techniques indicated that there was a low risk to human health [27]. In other studies, we have developed new techniques for quantitative health risk assessment, particularly with the Overall Risk Probability (ORP) method [29,30] which has been utilized in several health risk evaluations [31].

The aim of this study was to conduct a comprehensive evaluation of exposure to concentrations of BTX compounds in the ambient air for people in the service station environment as well as estimating the risk to health.

## 2. Methodology

### 2.1. Study Strategy

The strategy used in this research involves collection of data on BTX concentrations in the air of the service station environment and guideline values for human health from the scientific literature. The data sets were collated into different categories described as scenarios according to activity, location and description. Subsequently the BTX exposure data sets were used to develop Cumulative Probability Distribution (CPD) plots for each scenario. From the CPD plots, exposure at 50% (C_EXP50_) and 95% (C_EXP95_) cumulative probability levels were estimated to evaluate the Hazard Quotient (HQ) and Cancer Risk (CR). Overall Risk Probability for cancer was estimated for all the data points.

### 2.2. Sources of Data

The data sets that were utilized to evaluate the risk to human health were concentrations in air that can result in exposure through inhalation by humans in the service station environment. The data sets used to quantify the exposure to BTX were collected from an extensive search of the scientific literature using various search engines such as Google Scholar, Web of Knowledge, Web of Science and Science Direct. The source and nature of the datasets are summarized in Table 1. Each reference provided one or more sets of individual BTX measurements, with each set representing measurements for a sampling location, activity or job description. A number of data sets were available where only mean concentration were reported [2,7,24,25,32,33,34,35,36,37,38,39,40,41,42]. These were not included in the risk assessment exposure analysis. Since they cannot be combined and interpreted with the datasets on individual measurements.

### 2.3. Criteria for Data Selection

The health risk assessment is focused on evaluating exposure data on BTX concentrations in the ambient air of service station environments. The datasets were related to exposure through inhalation of BTX in the ambient air rather than uptake from other routes. Only data sets reported in the last 20 years from 1993 to 2013 (inclusive) were utilized to improve consistency between datasets. Data was reported in two forms:

(i) Original data on individual concentrations and mean concentrations of BTX. The mean data sets were not used since the way in which the means were derived were not consistent between datasets and the risk characterisation depends on specific exposure concentrations.
(ii) Original data on individual BTX concentrations in service station environments were utilized based on studies with similar chemical and analytic methods, samples were collected with similar techniques and studies done in the last 20 years (1993–2013).

### 2.4. Cumulative Probability Distribution (CPD) Plots

The data sets were used to produce the Cumulative Probability Distribution (CPD) plots to evaluate the exposures measured in the various service stations scenarios. Microsoft Excel was used in constructing CPD Plots from data collected from the investigations listed in Table 1. The CP% was calculated from:

P(%)= *(i/n+1)* × 100% (1)

where CP is cumulative probability (%), *i*, *i*th point and n, total number of data points.

The linear regression equations of the CPD plots for benzene, toluene and xylene were calculated between approximately 20%–80% of the Cumulative Probability since this represents the approximately linear part of the CPD plots when a normal distribution occurs.

### 2.5. BTX Exposure Limit

The exposure limits for exposure to BTX was obtained from online database and summarised in Table 2 [18,19,43,44,45,46,47].The exposure limits were for occupational exposure to BTX from various organisations such as European Commission, OSHA, NIOSH, ACIGH and SAOHS (Table 2) and Air Quality Guidelines (AQGs) from WHO, United Kingdom, Canada and European Union (Table 2).

**Table 2:** Occupational exposure limits and air quality guideline values for exposure to benzene, toluene and xylene.

Standards and Guidelines	Regulatory Body	Description	Benzene (µg/m^3^)	Toluene (µg/m^3^)	Xylenes (µg/m^3^)
Occupational Exposure Limits (OEL)	American Conference of Governmental Industrial Hygienists (ACGIH), USA	Threshold Limit Values (TLVs)	1600	75,000	435,000
		Short Term Exposure Limit (STEL)	8000	Not available	655,000
	Occupational Safety and Health Administration (OSHA), USA	Permissible Exposure Limit (PEL)	3250	750,000	435,000
		Short Term Exposure Limit (STEL)	16,250	Not available	655,000
	National Institute for Occupational Safety and Health (NIOSH), USA	Recommended Exposure Limit (REL)	325	375,000	435,000
		Short Term Exposure Limit (STEL)	3250	560,000	65,500
	South African Occupational Health and Safety (SAOHS), South Africa	Occupational Exposure limit (OEL)	1600	175,000	435,000
	European Directives 2000/39/EC and 97/42/EC (ED), European Union	Limit Value (LV) for occupational exposure	3250	Not available	Not available
Air Quality Guidelines (AQGs)	World Health Organisation (WHO), Global	1week (toluene)	Not available	260	
		24h (xylene)			480
	European Union Directives 2000/69/EC, European Union	Annual mean	5	Not available	Not available
	Expert Panel on Air Quality Standards (EPAQS), UK	Annual mean	16.25	Not available	Not available
	Alberta Ambient Air Quality Objective (AAAQO), Government of Alberta, Canada	1h (benzene)	30		
		24h (toluene; xylene)		400	700

Exposure evaluation of concentrations of BTX in the various scenarios were in occupational and non-occupational settings. Occupational exposure limits for 8 h—Time Weighted Average (TWA) were used to compare exposures to BTX for scenarios 1, 2, 3 and 4, while Air Quality Guideline (AQGs) were used to compare exposure to BTX in scenario 6. Because there was no stated guideline values for customers during refueling, Short Term Exposure Limit (STEL) for BTX (Table 2) were used to evaluate exposure in scenario 5.

### 2.6. Exposure Evaluation

#### 2.6.1. Background

The data sets were obtained from the publications listed in Table 1. Benzene, toluene and xylene concentration data were converted from mg/m^3^ and ppb to a uniform unit of µg/m^3^. The data sets for developing the CPD plots were categorized according to activity, location and occupation. Like data sets were grouped together as scenarios as described below.

#### 2.6.2. Scenario 1—Exposure of Service Station Attendants to BTX Concentrations in Air

This scenario was for service station attendants dispensing petrol to drivers in situations where there were no self-service facilities. Personal air samples were collected using air sampling pumps worn by the attendants during their work shift. The data sets used in this scenario were obtained from the following references and had the means and standard deviations for benzene (B), toluene (T) and xylene (X) as indicated: [3], B (910 ± 140); T (1580 ± 180); X (890 ± 110); [4], B (4 ± 1); T (241 ± 118); X (240 ± 122); [7], B (650 ± 470); T (730 ± 410); X (310 ± 160); [13], B (1081 ± 491); T (2180 ± 1100); X (1940 ± 2060).

#### 2.6.3. Scenario 2—Exposure to Concentrations of BTX in Air for Mechanics Repairing Petrol Pumps

This scenario was for exposure to ambient BTX concentrations as 8 h TWA for mechanics repairing and maintaining petrol dispensing pumps. Personal air samples w13ere collected by the air sampling pumps worn by the mechanics during work. The data sets used in this scenario were obtained from the reference and had the means and standard deviations for benzene (B), toluene (T) and xylene (X) as indicated: [12], B (233 ± 165); T (2218 ± 1736); X (553 ± 548).

#### 2.6.4. Scenario 3—Exposure of People to Concentrations of BTX in Air within the Service Stations

This scenario was for exposure to ambient BTX concentrations at various points within the forecourt of the service station. Air concentrations were measured using static air pumps close to petrol dispensing pumps and within the forecourt perimeter. Service station attendants are the most likely exposed group although, individuals using the service stations are also possibly exposed. The data sets used in this scenario were obtained from the following references and had the means and standard deviations for benzene (B), toluene (T) and xylene (X) as indicated: [1], B (4 ± 2); [5], B (29.7 ± 19.7); T (47.7 ± 27.4); X (28 ± 23); [8], B (10 ± 5); [9], B (14 ± 12); [10], B (91 ± 75); T (417 ± 390); [15], B (4 ± 3).

#### 2.6.5. Scenario 4—Exposure of Workers to Concentrations of BTX in Air in the Offices of Service Stations

This scenario covers people working in the offices of service stations who are exposed as a result of BTX concentration levels in the air. Air samples were taken inside the offices of the service stations using personal sampling pumps. The data sets used in this scenario were obtained from the reference and had the means and standard deviations for benzene (B), toluene (T) and xylene (X) as indicated: [4], B (5 ± 6); T (330 ± 393); X (157 ± 122).

#### 2.6.6. Scenario 5—Exposure to Concentrations of BTX in Air for Customers during Car Refueling

This scenario was for exposure of customers to ambient BTX concentrations during car refueling. Semipermeable Membrane Devices (SPMDs) were deployed on peoples clothing for a few minutes during vehicle refuelling. Sampling time was between 2–40 min. An average of 10 min was calculated in estimating the risk of health effects. The data sets used in this scenario were obtained from the reference and had the means and standard deviations for benzene (B), toluene (T) and xylene (X) as indicated: [11], B (1767 ± 1595); T (27,878 ± 28,337); X (957 ± 1235).

#### 2.6.7. Scenario 6—Exposure to Concentrations of BTX in Air for People External to the Service Stations

This scenario covers emission of BTX from the service stations to the immediate surroundings giving exposure to people living near service stations. BTX concentration data sets were obtained from the boundary to a distance of 5 to 300 m from the service stations. BTX air concentrations were collected using static air pumps at different positions from the boundary to the background level of the service stations. The data sets used in this scenario were obtained from the following references and had the means and standard deviations for benzene (B), toluene (T) and xylene(X) as indicated: [5], B (17 ± 3); [6], B (27 ± 38); T (23 ± 4); X (28 ± 23); [9], B (3 ± 2); T (57 ± 66); X (25 ± 27); [14], B (14 ± 17); [15], B (2 ± 1); [16], B (4 ± 3).

### 2.7. Risk Characterisation

#### 2.7.1. Background

The data sets for exposure to concentrations of BTX in the air were based on personal monitoring and from static sampling. Original data for individual concentrations were used to calculate the Hazard Quotient, Cancer Risk and Overall Risk Probability.

#### 2.7.2. Calculation of Lifetime Average Daily Dose (LADD)

The Lifetime Average Daily Doses (LADD) (µg/kg/day) for exposure to BTX concentrations in air through inhalation were calculated for occupational and non-occupational exposure using Equation (2):

LADD= *C_EXP_* × IR × EL × ED/BW × LT (2)

where C*_EXP_* is exposure concentration (µg/m^3^); IR, Inhalation Rate (m^3^/h); EL, Exposure Length (h/day); ED, the Exposure Duration (days); BW, Body Weight (kg) LT, Life Time (days). Exposure values for indoor and outdoor IR, EL ED and LT are presented in Table 3 [23,48]. The evaluations of these values with the different scenarios are shown in Table 3.

**Table 3:** Summary of USEPA standard default exposure factors [23,48].

Parameter	Unit	Default Value
Lifetime (LT)	years	70
Body Weight (BW)	kg	70
Exposure Length (EL)	h/day	8 (workers)4 (outdoor)
Exposure Duration (ED)	years	25 (commercial/industrial)/30 (residential)
Inhalation Rate (IR)	m^3^/h	0.83 (indoor)1.4 (outdoor)

Notes: LT = 7 days/week × 52 weeks/year × 70 years = 25,480 days (Scenario 1 to 6); ED = 5 days/week × 48 weeks/year × 25 years = 6000 days (Scenario 1, 2 and 4); ED = 7 days/week × 52 weeks/year × 25 years = 9100 days (Scenario 3); ED = 1 days/week × 52 weeks/year × 30 years = 1560 days (Scenario 5); ED = 7 days/week × 52 weeks/year × 30 years = 10,920 days (Scenario 6); IR = 1.4 m^3^/h (Scenario 1, 2, 3, 5 and 6); IR = 0.83 m^3^/h (Scenario 4).

#### 2.7.3. Calculation of Hazard Quotient (HQ)

**Figure 1:**
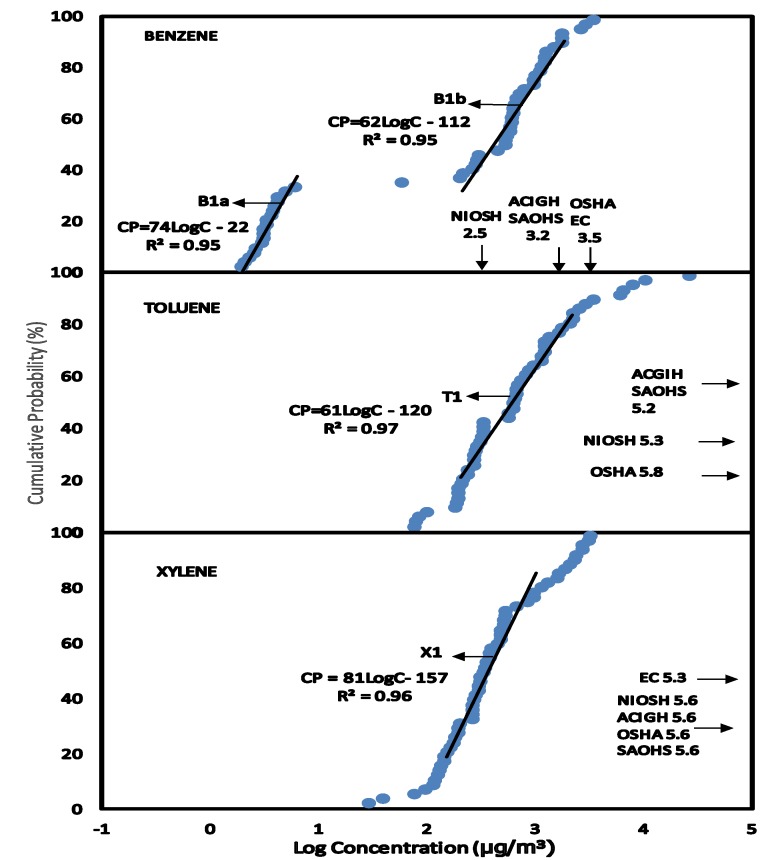
Scenario 1, CPD plots for exposure of service station attendants to concentrations of benzene, toluene and xylene in the air of service stations as measured by personal air sampling pumps.

**Figure 2:**
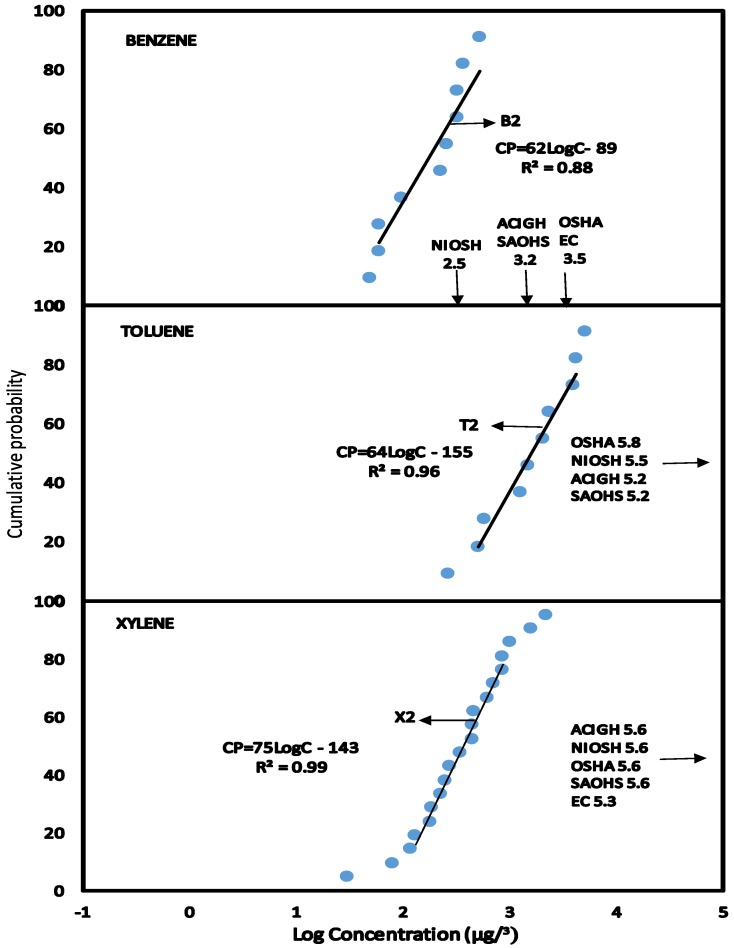
Scenario 2, CPD plots for exposure to mechanics repairing and maintaining fuel pumps to concentrations of benzene, toluene and xylene in air as measured by personal air sampling pump.

**Figure 3:**
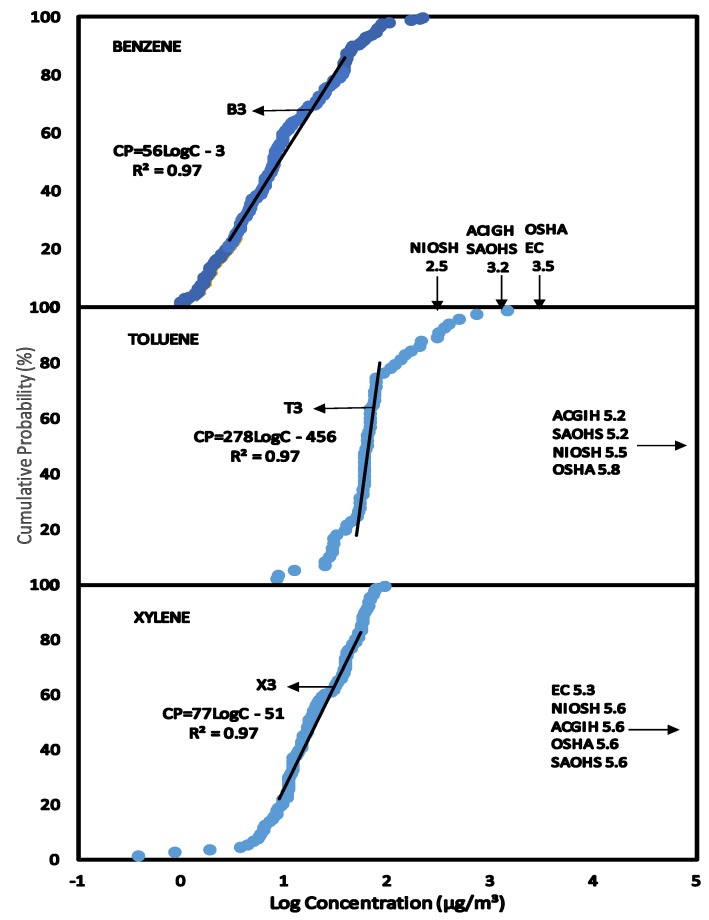
Scenario 3, CPD plots for exposure to concentrations of benzene, toluene and xylene in the air for people operating within the forecourt perimeter of the service stations as measured by static air sampling pumps.

**Figure 4:**
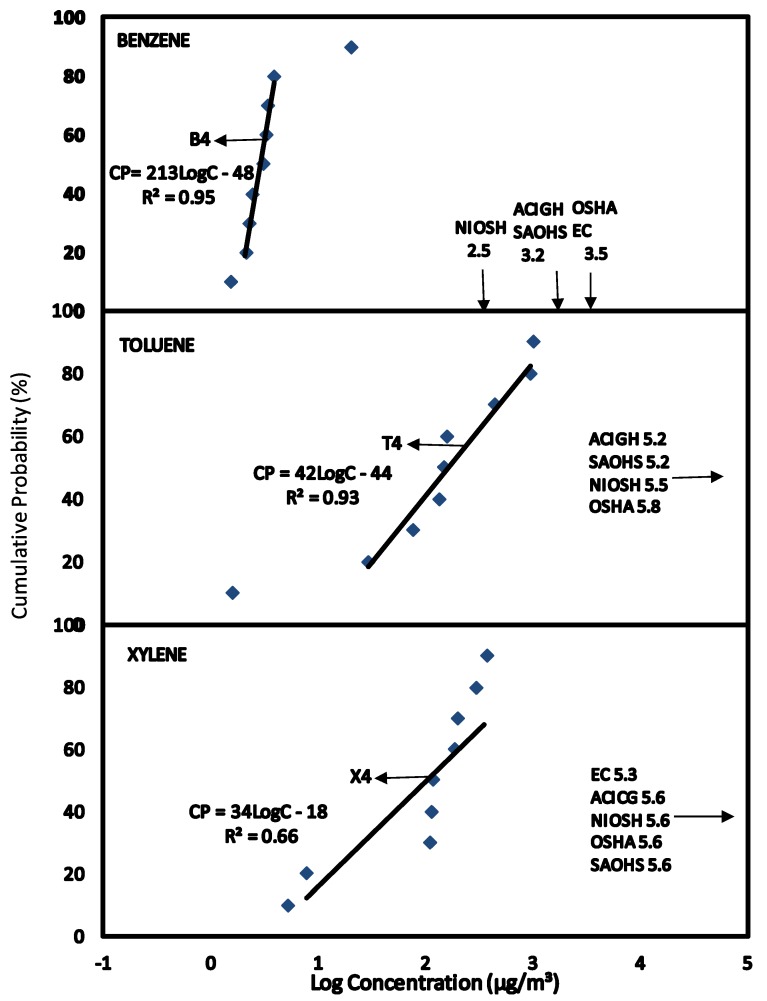
Scenario 4, CPD plots for exposure of workers to concentrations of benzene, toluene and xylene in the air of offices of service stations as measured by personal air sampling pump.

**Figure 5:**
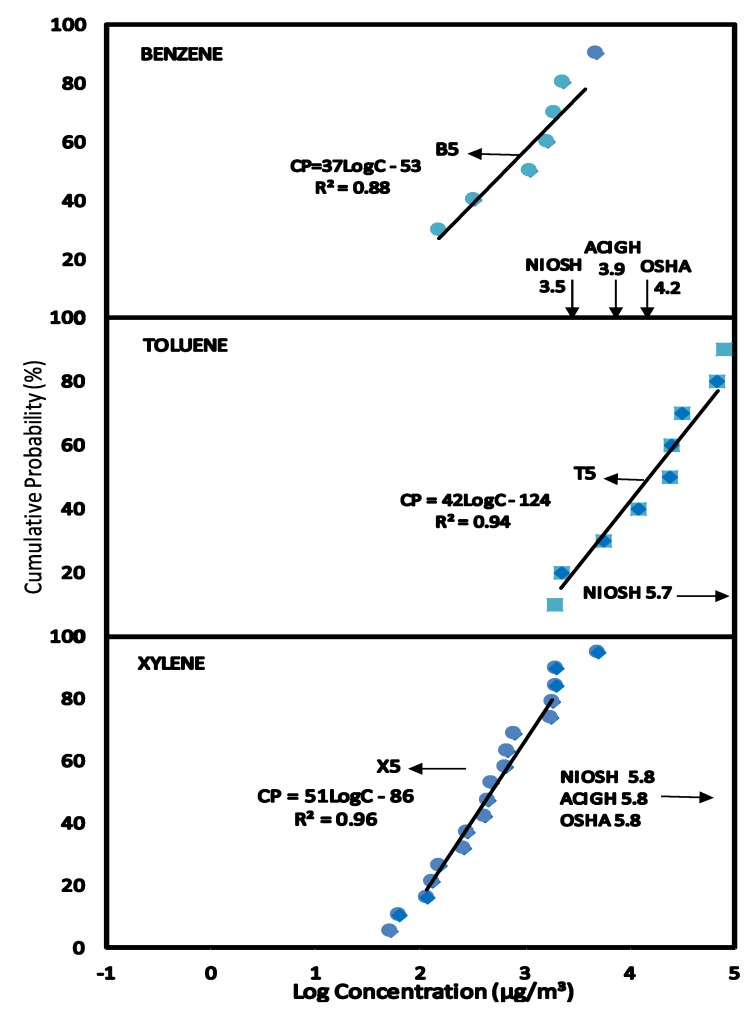
Scenario 5, CPD plots for exposure to customers to concentrations of benzene, toluene and xylene during car refueling as measured by deploying Semipermeable Membrane Devices (SPMDs) on peoples clothing for a few minutes during vehicle refueling.

**Figure 6:**
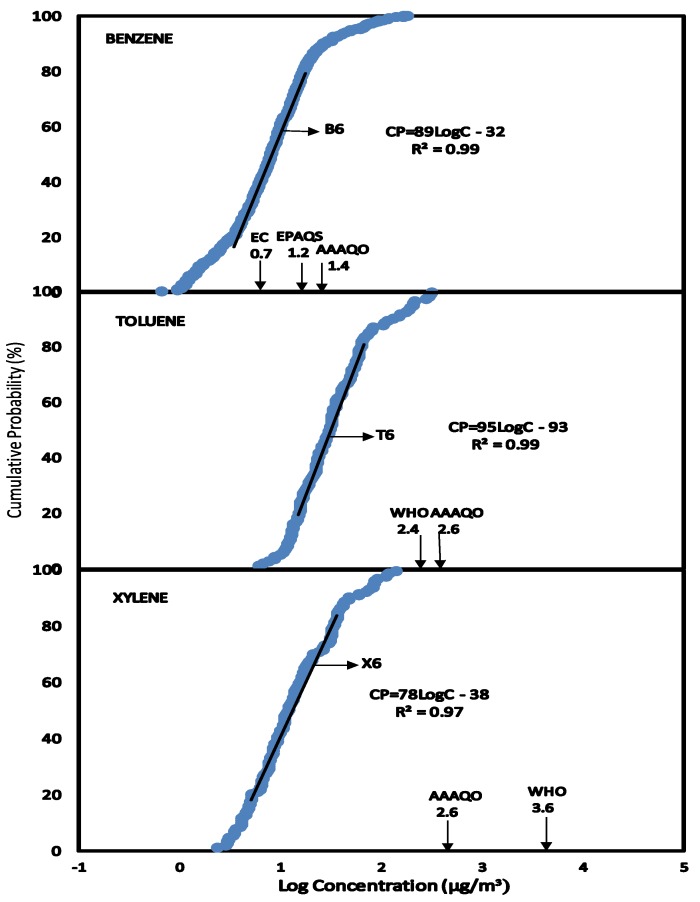
Scenario 6, CPD plots for exposure to people outside the service stations to concentrations of benzene, toluene and xylene from the service stations as measured by static air sampling pumps.

The HQ method of risk characterisation was used to estimate the non-cancer risk to human health on exposure to BTX concentrations. The Reference Dose (RfD) derived for BTX presented in Table 4 were used to estimate the HQ from exposure to BTX in the various scenarios. The BTX exposures were estimated at the median level (C_EXP50_) which represents the main group of individuals and the 95% level (C_EXP95_) representing the highest exposed group in the population. This highly exposed group occurs at a level of 5% in the population and the median group represents over 50% in the population. BTX exposure concentrations at C_EXP50_ and C_EXP95_ were obtained from the CPD plots in Figure 1, Figure 2, Figure 3, Figure 4, Figure 5 and Figure 6 (see Section 3—Results and Discussion) and converted to LADD using Equation (2). HQ was obtained as a ratio of exposure to BTX as LADD to the Reference dose (RfD) as shown in Equation (3):

HQ = LADD/RfD (3)

where HQ is the Hazard Quotient; LADD, lifetime average daily dose (µg/kg/day); RfD, reference dose (µg/kg/day).

**Table 4:** Slope Factor for benzene [23]; Reference Dose for benzene, toluene and xylene *.

Chemical	Inhalation Reference Dose (RfD) (mg/kg/day)	Inhalation Slope Factor (SF) (mg/kg/day)^-1^
Benzene	0.00855	0.0273
Toluene	1.4	Not available
Xylene	0.029	Not available

Note: * The Risk Assessment Information System [49].

#### 2.7.4. Calculation of Cancer Risk

According to USEPA, cancer risk is expressed as excess risk of developing cancer over a lifetime of exposure. The Cancer Risk (CR) on exposure to benzene through inhalation was quantitatively estimated at C_EXP50_ and C_EXP95_ by converting to LADD using Equation (4). CR for toluene and xylene could not be calculated because a slope factor value is not available for these compounds. Slope factor for benzene is given in Table 4. The Cancer Risk (CR) was calculated for exposure to benzene using Equation (4):

Cancer Risk = LADD (µg/kg/day) × SF (µg/kg/day)^-1^ (4)

where SF is slope factor for benzene.

#### 2.7.5. Estimation of Overall Risk Probability (ORP)

The ORP method is based on the use of the overall risk probability curve. The ORP curve is the plot of CP exposure exceedence values against the corresponding CP values for dose-adverse effects. A detailed description of overall risk probability in risk assessment has been discussed previously [30]. The calculated adverse effect CP plot was obtained by using Equation (4).

## 3. Results and Discussion

### 3.1. Scenario 1—Exposure of Service Station Attendants to BTX Concentrations in Air

Figure 1 is the CPD plots of benzene (B1–B2), toluene (T1–T2) and xylene (X1–X2) concentrations in air of service stations for attendants wearing air sampling pumps during work. From the linear equations obtained for BTX exposures (Figure 1), the slopes for benzene plots were 62 and 74, the slopes of toluene and xylene plots were 61 and 81 respectively. This indicates a similar pattern of distribution with all the BTX compounds.

The exposure to BTX concentrations in air in this scenario (Figure 1) was compared to NIOSH REL, ACIGH TLV, OSHA PEL, EC LV and SAOHS OEL. At C_EXP50_ and C_EXP95_, exposures to toluene and xylene were lower than the exposure limits. In addition, exposure to benzene at C_EXP50_ was higher than NIOSH REL but lower than ACIGH TLV, OSHA PEL, EC LV and SAOHS OEL. However, at C_EXP95_ exposure to benzene in air for service station attendants was higher than NIOSH REL, ACIGH TLV, EC LV and SAOHS but lower than OSHA PEL.

The CPD plots show diagrammatically the difference in the concentrations of benzene to toluene and xylene. From the lowest point to C_EXP40_, benzene concentrations (B1a) in air was about 30 to 100 times lower than benzene concentrations from C_EXP40_ to C_EXP95_ (B1b). However not much difference was observed for toluene and xylene concentrations. A major factor responsible for the difference in concentrations was the efficiency in the use of Stage 1 and 2 Vapor Recovery Systems (VRS) in some of the service stations [4] as compared to service stations without the use of VRS.

The ratio of the concentrations of benzene for service stations with VRS to service stations without was quantified as the Relative Presence (RP). The estimation of RP was based on average levels of BTX in the service stations that use Stage 1 and 2 VRS to service stations that did not utilize Stage 1 and 2 VRS:

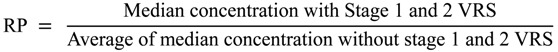
(5)


The RP for benzene, toluene and xylene were 0.004, 0.1 and 0.2 respectively. This was plotted against the boiling points of BTX to evaluate the influence of this factor. The linear equation for the plot was calculated as:

RP = 0.0032BP - 0.26, *R^2^* = 0.99 (6)

This RP in the air increases with the boiling point and is a reflection of the volatility of the compounds. The use of VRS results in benzene having the lowest concentration in the ambient air of the service stations since it is removed with the VRS.

### 3.2. Scenario 2—Exposure to Concentrations of BTX in Air for Mechanics Repairing Petrol Pumps

The concentration levels of benzene (B5), toluene (T5) and xylene (X5) collected during repair of petrol dispensing pumps by mechanics wearing personal sampling pumps in the service stations were plotted as CPD plots in Figure 2. The linear regression equations obtained from the CPD plots of benzene, toluene and xylene have almost identical slopes 62 and 64 and 75 respectively indicating similar distribution patterns.

The exposure data were used to compare the occupational exposure limits as shown in Table 2. At C_EXP50_ and C_EXP95_, none of the exposure values for toluene and xylene exceeds NIOSH REL, ACIGH TLV, OSHA PEL, SAOHS OEL, EC LV. At C_EXP50_ the concentration of benzene in air were below NIOSH REL, ACIGH TLV, OSHA PEL, SAOHS OEL and EC LV. However, the data points at C_EXP95_ as shown in Figure 5 have some concentrations of benzene higher than NIOSH REL but lower than ACIGH TLV, SAOHS OEL, EC LV and OSHA PEL.

### 3.3. Scenario 3—Exposure of People to Concentrations of BTX in Air within the Service Stations

The CPD plots for benzene (B2), toluene (T2) and xylene (X2) concentrations in air within the service stations forecourt perimeter are shown in Figure 3. Linear regression equations were obtained for each compound as shown in the CPD plots (Figure 3) with correlation coefficients (R^2^) of 0.97 indicating a high linearity of distribution of the data.

The exposures to BTX (Figure 3) were compared to the exposure limits in Table 2. At C_EXP50_, and C_EXP95_, all exposures to benzene, toluene and xylene plots were lower than NIOSH REL, ACIGH TLV, OSHA PEL, SAOHS OEL and EC LV.

### 3.4. Scenario 4—Exposure of Workers to Concentrations of BTX in Air in the Offices of Service Stations

The concentration levels of benzene (B3), toluene (T3) and xylene (X3) in the offices of service stations were plotted as CPD plots in Figure 4. None of the BTX exposures in the offices of the service stations exceeds the exposure limits in Table 2 at C_EXP50_ and C_EXP95_ (Figure 4). From the linear regression equations, xylene and toluene have almost identical slopes of 34 and 42 respectively which are quite different from the slope obtained for benzene (213, see Figure 4). This indicates that the concentrations of toluene and xylene were distributed over a wider range than benzene.

### 3.5. Scenario 5—Exposure to Concentrations of BTX in Air for Customers during Car Refueling

The concentration levels of benzene (B5), toluene (T5) and xylene (X5) collected during customers refueling of cars in the service stations were plotted as Figure 5. Linear equations were obtained from the CPD plots of benzene, toluene and xylene.

Exposure to BTX for customers during car refueling were compared with the short term exposure limit (STEL) for NIOSH, OSHA and ACIGH since there is no guideline for such situations. At C_EXP50_ and C_EXP95_, xylene was lower than NIOSH, OSHA and ACIGH STEL, while toluene was lower than NIOSH. However, at C_EXP50_ benzene was higher than the STEL of NIOSH, OSHA and ACIGH. At C_EXP95_ benzene was higher than NIOSH STEL but lower than the STEL of ACIGH and OSHA. Although the concentrations of BTX in this scenario was very high as compared to the others, however, the exposure is only for a short time for less than an hour and probably about once in a week.

### 3.6. Scenario 6—Exposure to Concentrations of BTX in Air for People External to the Service Stations

Ambient concentration levels of benzene, toluene and xylene from the boundary of the service stations to distances of approximately 300 meters were plotted as CPD plots B4, T4 and X4 as shown in Figure 6. From the CPD plots, the linear equations and correlation coefficients were obtained.

The exposure data were used to compare the Air Quality Guidelines (AQG) presented in Table 2. At C_EXP50_ and C_EXP95_, none of the exposures for toluene and xylene exceeds the AQG for WHO and AAAQO Canada. At C_EXP50_ benzene concentration levels were high than AQG for European Commission but lower than AQG for United Kingdom and AAAQO Canada. However, at C_EXP95_, concentrations of benzene were above both the AQG for United Kingdom Canada and European Commission.

### 3.7. Risk Characterization with HQ, CR and ORP

#### 3.7.1. Hazard Quotient (HQ) Calculated at C_EXP50_ and C_EXP95_

In comparing concentrations of benzene, toluene and xylene in the air of service stations to the OEL and AQGs, concentrations of toluene and xylene were below OEL and AQGs (Table 2) for all Scenarios. Therefore, further evaluation using the LADD for toluene and xylene were not carried out. The calculated LADD at C_EXP50_ and C_EXP95_ for exposure to benzene was used in estimating the HQ (Equation (3)) and the results were summarized in Table 5.

The HQ_50_ and HQ_95_ for lifetime exposure to benzene in scenario 3, scenario 4, scenario 5 and scenario 6 were <1. This result suggest minimal risk to the majority of the population in the exposure scenarios. HQ_50_ and HQ_95_ for lifetime exposure to benzene for service station attendants (scenario 1) and mechanics repairing petrol dispensing pumps (scenario 2) were >1 indicating possible adverse health effects.

**Table 5:** Calculated LADD, HQ and CR per 10^6^ at 50CP and 95CP for lifetime exposure to benzene concentrations in air through inhalation.

Scenario	LADD_50_ (µg/kg/day)	LADD_95_ (µg/kg/day)	HQ at LADD_50_	HQ at LADD_95_	CR per 10^6^ at LADD_50_	CR per 10^6^ at LADD_95_	CR per 10^6^ Estimated by ORP
Scenario 1 (Service station attendants)	12	66	1.4	7.8	340	1800	670
Scenario 2 (Mechanics)	9.0	24	1.1	2.8	240	650	320
Scenario 3 (Inside service stations)	0.23	2.3	0.027	0.27	6.0	62	170
Scenario 4 (Workers in offices)	0.069	0.72	0.0081	0.084	2.0	21	100
Scenario 5 (Customers refuelling)	0.29	1.0	0.034	0.12	8.0	28	90
Scenario 6 (Outside service stations)	0.35	2.2	0.041	0.26	10.0	61	160

#### 3.7.2. Cancer Risk Calculated at C_EXP50_ and C_EXP95_

The excess CR was calculated for exposure through inhalation for benzene concentrations in the air of service stations for all the scenarios at C_EXP50_ and C_EXP95_ and the results were presented in Table 5. The estimated CR for a lifetime exposure to concentrations of benzene within and outside the service stations for majority of the population (C_EXP50_) and the high exposed group (C_EXP95_) in scenario 1 to 6 were 340 to 180, 240 to 650, 6 to 62, 2 to 21, 8 to 28 and 9 to 61 per 10^6^ respectively. The result suggest potential cancer risk for lifetime exposure to benzene in the various scenarios but at different levels. The excessive cancer risk for scenario 3, 4, 5 and 6 are effectively the same and they are very low as compared to scenario 1 and 2. However, the highest cancer risk was observed for service station attendants.

#### 3.7.3. Overall Risk Probability (ORP)

**Figure 7:**
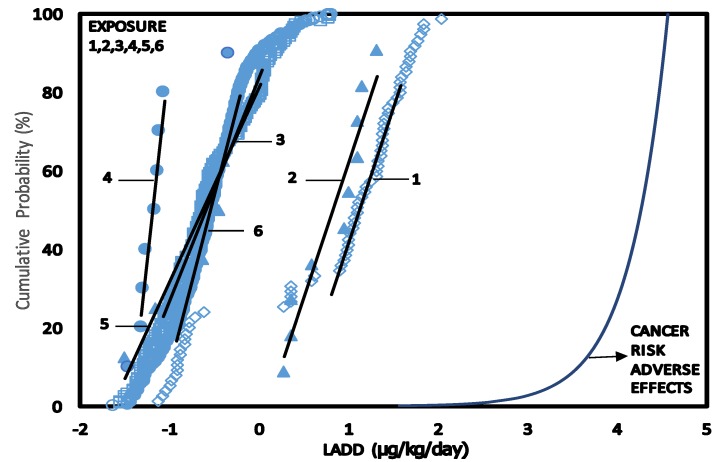
CPD plots lifetime exposure to benzene concentrations in air of service station (LADD) for scenarios 1–6 and Adverse Effects.

**Figure 8:**
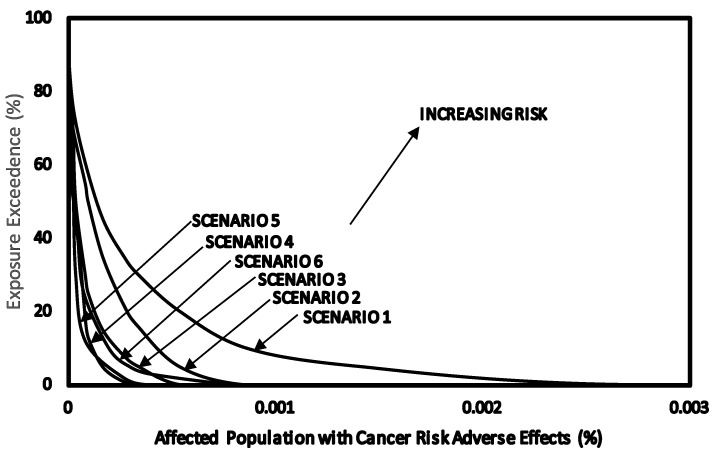
Overall risk probability for cancer as a result of exposure to benzene concentrations in air of service station environments.

The overall risk probability for cancer was estimated as the percentage of cancer risk for exposure to benzene in the various scenarios. The exposure exceedence values as percentage were calculated and plotted against the percentage of affected population to obtain an ORP curve for scenarios 1 to 6. The CPD plots for scenarios 1 to 6 together with the dose—cancer response curves are shown in Figure 7. The ORP plots are shown in Figure 8.The area under the ORP curves were calculated to obtain values of Overall Risk Probability of 0.067% (670 per 10^6^) for service station attendants (scenario 1), 0.032% (320 per 10^6^) mechanics repairing fuel dispensing pumps (scenario 2), 0.017% (170 per 10^6^) of exposed population inside (scenario 3), 0.015% (150 per 10^6^) of exposed outside service stations (scenario 6), 0.010% (100 per 10^6^) of workers in the office of service stations (scenario 4) and 0.009% (90 per 10^6^) customers during car refuelling (scenario 5).

The difference between the ORP method and single point methods such as HQ and CR in risk characterisation, is that HQ and CR were calculated for high exposed group (C_EXP95_) and the main group of individuals (C_EXP50_) in the population, while in using ORP all of the exposed population were taken into consideration.

## 4. Conclusions

Of the BTX compounds, benzene is the compound of most concern because levels higher than the exposure limits were observed in some scenarios. The time spent during work and the proximity of emission sources is a major contributor to BTX exposure. Service station attendants and workers maintaining petrol dispensing pumps were at risk of been exposed to relatively high levels of benzene in concentrations ranging from 1.9 to 2900 µg/m^3^ and 51 to 540 µg/m^3^ respectively. Although, appreciably high concentrations of benzene were observed with customers refueling cars (150 to 4900 µg/m^3^). The vapour recovery systems (VRS) reduced the level of exposure to benzene to levels of minimal concern.

Customers had only about 10 min of exposure per week which results in HQ_50_ < 1 and HQ_95_ < 1. The lifetime exposure to benzene for service station attendants (scenario 1) and mechanics repairing petrol dispensing pumps (scenario 2) had HQ_50_ > 1 and HQ_95_ > 1 suggesting possible adverse health effects. The lifetime exposure to benzene for scenario 3, scenario 4, scenario 5 and scenario 6 had HQ_50_ < 1 and HQ_95_ < 1 in all scenarios. Also, HQ_50_ < 1 and HQ_95_ < 1 was estimated for toluene and xylene in all scenarios suggesting minimal risk to the majority of the population in the exposure scenarios.

The results indicated low risk from lifetime exposure to benzene concentrations in the offices of service stations (1.6 to 20 µg/m^3^), within (1.0 to 220 µg/m^3^) and outside (0.71 to 190 µg/m^3^) the service stations. The estimated CR suggests that chronic exposure to benzene concentrations in air for all the scenarios poses a potential cancer risk. The highest cases of CR was observed for service station attendants ranging from 340 to 1800 in 1,000,000 exposed population. The values obtained using the ORP method suggest that service station attendants are at more risk to adverse health effects than the other exposure Scenarios.
